# Diagnostic use of infrared thermography in a patient with chronic pain following electrocution: a case report

**DOI:** 10.4076/1752-1947-3-8992

**Published:** 2009-09-09

**Authors:** John Jarrell, Chris Spanswick

**Affiliations:** 1 Department of Obstetrics and Gynecology, University of Calgary, Calgary, AB, Canada; 2 Calgary Health Region Chronic Pain Centre, Calgary, AB, Canada

## Abstract

**Introduction:**

Survival after severe electrocution is uncommon but chronic pain after such trauma is rare. We present a case report of an individual in whom the only modality providing objective evidence of pain related injury was infrared thermography.

**Case presentation:**

A 35-year-old Caucasian woman presented to the Calgary Health Region Chronic Pain Centre with severe pain in her left hand and foot following electrocution. All previous clinical and neurological testing had been normal. Infrared thermography demonstrated a significant reduction in temperature in the regions affected on her left hand and foot. Pain was reduced with the use of pregabalin but without changes to thermal differences in the affected limbs.

**Conclusion:**

It would appear from this case report that infrared thermography may be of use in the documentation of abnormalities associated with chronic pain following survival after electrocution. Pregabalin may be of benefit in pain reduction after electrocution.

## Introduction

Survival after severe electrocution is uncommon but chronic pain following such trauma is rare. In Calgary, Alberta, Canada, a population study indicated the rate of severe electrical trauma was 2.4 per million population annually [1]. We present a case of low voltage electrocution in a patient where the use of clinical infrared thermography was helpful in documenting abnormalities associated with chronic pain, although the thermographic abnormalities did not change despite significant reductions in pain following treatment with pregabalin.

## Case presentation

The patient was a 35-year-old Caucasian woman who presented to the Calgary Health Region Chronic Pain Centre with severe pain in her left hand and foot. On 4 November 1994, she has been caught between an electrical stove and refrigerator and electrocuted for an unknown period of time with 220 V. The electrocution was associated with a 'no let go' contact with the power source until the door of the electric stove was pulled off. There was no loss of consciousness but the patient could not move from the floor for approximately 30 minutes. She reported that her muscles went into a severe spasm and she felt shaken and unwell. There were no burns on the skin or entry or exit wounds. She went to the emergency room where there were no objective signs of injury. An electrocardiogram demonstrated no evidence of myocardial ischemia or arrhythmia.

The patient later developed chronic pain described as a stabbing sensation in her left hand and left foot radiating to her elbow and knee, respectively. The left leg felt 'deadened' for about a year. There was an increased pain experience associated with all activities.

Neurological consultation was sought in 1995 and the findings included normal cranial nerves, and a normal sensory and motor examination. Deep tendon reflexes were symmetrical and there were down-going plantar responses. Tests of gait and coordination were normal. The neurologist made the diagnosis of 'dysesthetic neuropathic pain following electrical injury, possibly associated with demyelination in the spinal cord similar to a person who survived lightning strike'.

Since the pain persisted, the patient saw another neurologist in 1999 who performed an electromyogram (EMG) and nerve conduction studies. Both medial and ulnar nerves as well as the tibial and the left peroneal nerve were tested. All sensory and motor responses were completely normal. The opinion was that there was no evidence of a peripheral nerve dysfunction. Magnetic resonance imaging (MRI) of the cervical spine was carried out in June 1999, and was normal. Somatosensory evoked potentials were performed in August 1999 and these were normal in relation to the bilateral median and tibial nerves. The opinion was that the pain was associated with the electrocution but the pathophysiology remained elusive.

The patient entered the Calgary Health Region Chronic Pain Centre in June 2003. She confirmed persistent stabbing pain in her left hand and foot with essentially no change since the event. No general abnormalities in the clinical neurological examination were noted but the patient had evidence of increased tactile cutaneous allodynia in the anterior abdominal wall that was substantially higher on the left and extending upward to the rib cage down to the lower border of the L1 dermatome.

Clinical thermography was undertaken using the med2000 IRIS system provided by Meditherm Inc., with WinTES software that permits a computer to communicate with the Meditherm infrared camera (Meditherm Inc., Beaufort, NC, USA). Standardized protocols for image capture and measurement of the images were used.

**Figure 1:**
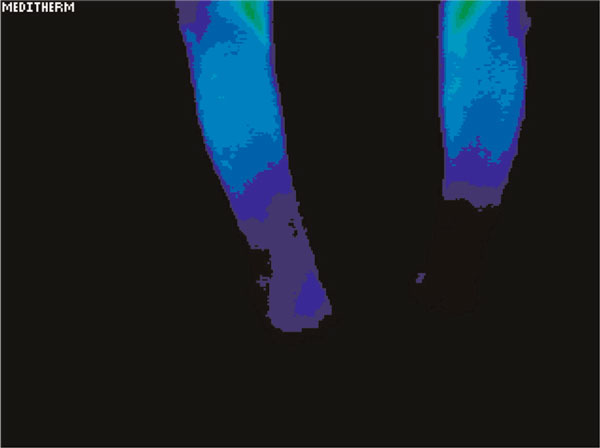
**Significant cooling of the left hand relative to the right**. The date of this test was 21 June 2003.

**Figure 2:**
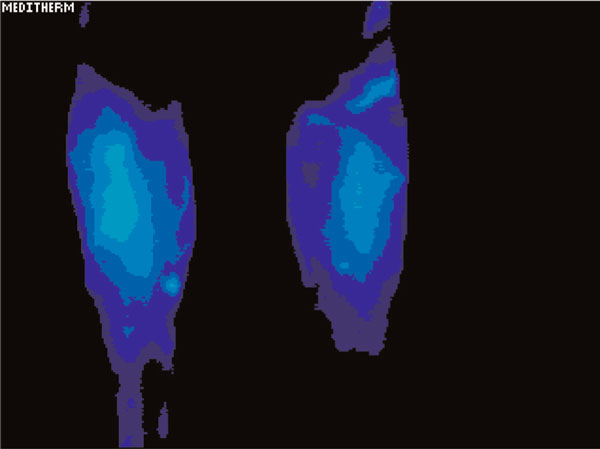
**Relative cooling of the left foot on the affected side**. The date of this test was 21 June 2003.

**Figure 3:**
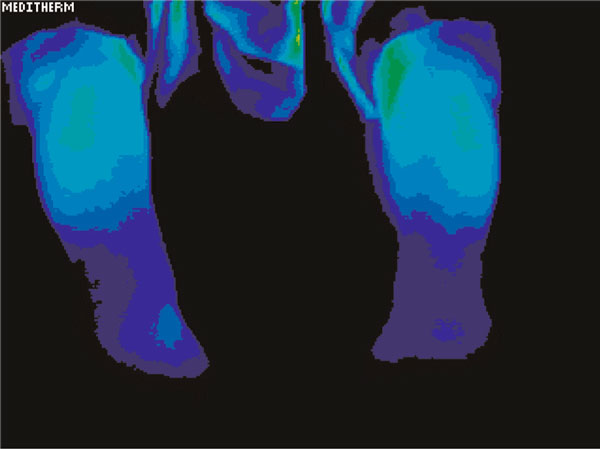
**Persistent relative cooling of the dorsum of the left hand on 1 May 2006**.

**Figure 4:**
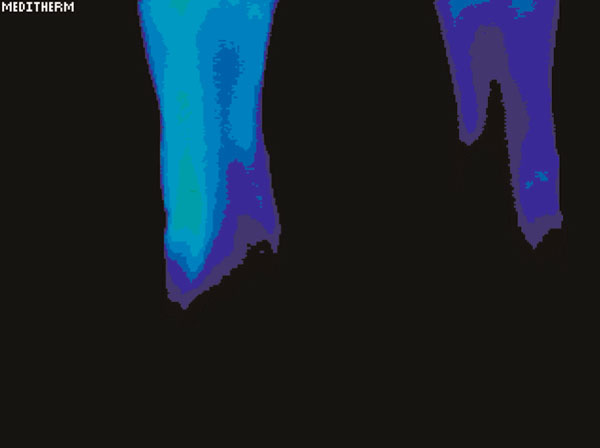
**Persistent significant cooling in the left foot on 1 May 2006**.

Testing showed a significant difference in temperature involving the left hand and foot as well as the lower arm and lower leg in the regions of discomfort. The maximally different temperatures are recorded in Table 1. Figure 1 demonstrates the relative cooling of the left hand relative to the right. The date of this test was 21 June 2003. Figure 2 demonstrates a relative cooling on the affected side in the left foot. Figure 3 demonstrates a similar reduction of the difference in temperature in the dorsum of the left hand on 1 May 2006. In Figure 4, there is still a significant difference in temperature in the left foot on 1 May 2006.

**Table 1:** Temperature measurements in sites of severe chronic pain and differences from contralateral sites (D°T) over the course of three years

Site	Date	Average Temp Right	Average Temp Left	D°T
Arm prone	2003 06 21	30.67	29.67	1.0°C
Leg anterior	2003 06 21	29.97	29.03	0.94°C
Arm prone	2006 05 01	28.43	27.52	0.91°C
Leg anterior	2006 05 01	25.44	24.35	1.09°C

D°T: Delta temperature or the difference in temperature measured when a site is compared with an identical contralateral site.

The patient was treated with pregabalin, 600 mg/day which was later reduced to 300 mg/day because of weight gain. On this regimen, there was a reduction in pain from 8/10 to 4–5/10 using self-reported pain scales. This reduction in pain was noted within weeks. Although there was a reduction in pain after the administration of pregabalin, there were no differences in pain measurements following the reduction in reported pain.

## Discussion

Thermography is a clinical test that measures the changes in cutaneous temperature in response to the physiological state of an individual [2]. As temperature from the body is eliminated as infrared energy, infrared thermography is ideal to depict contralateral disparities, indicating altered physiology or pathological states. Since there is a high degree of thermal symmetry in the normal body, subtle abnormal temperature asymmetries can be easily identified. Delta T is a measure of the temperature difference between similar sites of the body and a difference greater than 1ºC is accepted as abnormal. This report is of interest as it documents an abnormality in the cutaneous temperature as determined by infrared digital imaging when all other diagnostic testing did not indicate an abnormality. In this patient, despite a severe electrocution, there were no abnormalities in the clinical neurological examination, MRI scan, nerve conduction studies or neural evoked potentials, the traditional investigations of nerve injury.

Also of interest is the fact that the patient experienced a reduction in pain with the use of pregabalin. We believe this is the first report of a reduction in pain that is secondary to severe electrocution, which is usually a fatal event. Of interest, however, during the period of clinical improvement, there was no change in the differential temperature of the left hand and foot. This may indicate that, although central processing of pain can be reduced, the neural injury, possibly of the sympathetic system, is irreversible. Pregabalin has proved effective for reducing various sorts of neuropathic pain such as spinal cord injury, post-herpetic neuralgia as well as seizures, and it is FDA approved for use with fibromyalgia [3,4]. To our knowledge, this is the first time it has been shown to reduce chronic pain originating from electrocution.

Electrical current at low frequency (below microwave current) becomes distributed so that the electrical field strength in nearly perpendicular to the path of the current and the density distribution depends on the relative electrical conductivity of various tissues and the frequency of the current. In experimental animals, the major arteries and nerves experience the largest current density because of the higher conductivity [5]. Although some authors have reported that the primary nerve target is the myelinated nerve, it would appear that the principal injury in our patient was to the sympathetic nerves [6].

The use of thermography for similar apparent neuropathic pain has been identified for orofacial pain [7] and for complex regional pain syndromes [8]. It would appear from this case report that thermography may also have utility in the documentation of abnormalities associated with chronic pain following electrocution.

## Conclusion

This case report serves to indicate that infrared thermography may be of use in documenting abnormalities of peripheral nerves when other traditional modalities do not indicate abnormal neural function.

## Patient's perspective

It has now been 14 years since my electrocution. Having to live with a subjective injury has proven to be very difficult. I have seen several doctors and specialists who have conducted many tests, with no objective results. The thermography test was the first time that there was 'proof' of my pain. Though I have had a reduction in the pain level due to the use of pregabalin, I am still dealing with issues of chronic pain.

## Abbreviations

D°T: delta temperature or the difference in temperature measured when a site is compared to an identical contralateral site; EMG: electromyogram; MRI: magnetic resonance imaging.

## Consent

Written informed consent was obtained from the patient for publication of this case report and any accompanying images. A copy of the written consent is available for review by the Editor-in-Chief of this journal.

## Competing interests

The authors declare that they have no competing interests.

## Authors' contributions

JJ undertook the infrared thermography and was the major contributor to the manuscript. CS treated the patient with pregabalin and read and approved the final manuscript.
